# A Novel MRI Diagnosis Method for Brain Tumor Classification Based on CNN and Bayesian Optimization

**DOI:** 10.3390/healthcare10030494

**Published:** 2022-03-08

**Authors:** Mohamed Ait Amou, Kewen Xia, Souha Kamhi, Mohamed Mouhafid

**Affiliations:** 1 School of Electronics and Information Engineering, Hebei University of Technology, Tianjin 300401, China; mohamed.mouhafid@outlook.com; 2 State Key Laboratory of Reliability and Intelligence of Electrical Equipment, Hebei University of Technology, Tianjin 300130, China; souhakamhi@outlook.com

**Keywords:** MRI diagnosis, brain tumor classification, CNN, Bayesian Optimization

## Abstract

Brain tumor is one of the most aggressive diseases nowadays, resulting in a very short life span if it is diagnosed at an advanced stage. The treatment planning phase is thus essential for enhancing the quality of life for patients. The use of Magnetic Resonance Imaging (MRI) in the diagnosis of brain tumors is extremely widespread, but the manual interpretation of large amounts of images requires considerable effort and is prone to human errors. Hence, an automated method is necessary to identify the most common brain tumors. Convolutional Neural Network (CNN) architectures are successful in image classification due to their high layer count, which enables them to conceive the features effectively on their own. The tuning of CNN hyperparameters is critical in every dataset since it has a significant impact on the efficiency of the training model. Given the high dimensionality and complexity of the data, manual hyperparameter tuning would take an inordinate amount of time, with the possibility of failing to identify the optimal hyperparameters. In this paper, we proposed a Bayesian Optimization-based efficient hyperparameter optimization technique for CNN. This method was evaluated by classifying 3064 T-1-weighted CE-MRI images into three types of brain tumors (Glioma, Meningioma, and Pituitary). Based on Transfer Learning, the performance of five well-recognized deep pre-trained models is compared with that of the optimized CNN. After using Bayesian Optimization, our CNN was able to attain 98.70% validation accuracy at best without data augmentation or cropping lesion techniques, while VGG16, VGG19, ResNet50, InceptionV3, and DenseNet201 achieved 97.08%, 96.43%, 89.29%, 92.86%, and 94.81% validation accuracy, respectively. Moreover, the proposed model outperforms state-of-the-art methods on the CE-MRI dataset, demonstrating the feasibility of automating hyperparameter optimization.

## 1. Introduction

**Figure 1:**
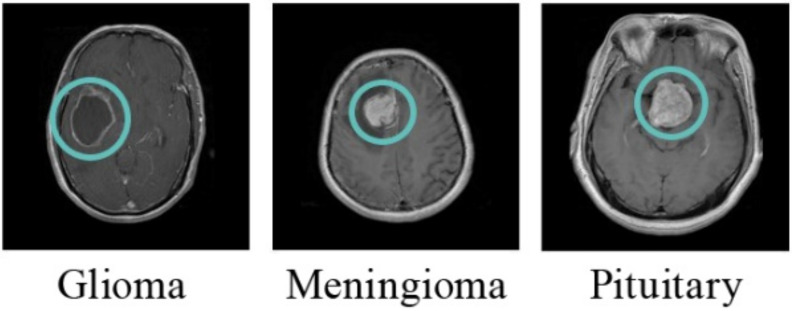
The most common forms of brain tumor (the tumors are localized inside a green circle).

In medical science, brain tumor is one of the most feared diseases. In 2016, it was the most typical cause of cancer-related death among children (ages 0–14) in the United States [1]. A brain tumor can be defined as an abnormal growth in brain cells. The most frequent forms of brain tumors include Meningioma, Glioma, and Pituitary (shown in Figure 1). The malignancy levels of these tumors differ from one another. Glioma is the most prominent malignant brain tumor that occurs in the tissues of the glia and the spinal cord. While Meningioma is a benign tumor (slow-growing tumor) that forms on the area that protects the brain and spinal cord (the membrane) [2,3,4,5]. Pituitary forms in the pituitary gland region. It is also a benign tumor, but unlike Meningioma, it may lead to other medical damage [4,5].

Brain MRI image is specifically used to detect tumor and tumor progress. The MRI image offers detailed information on brain structure more than CT or ultrasound image. Radiologists identify brain tumors during the analysis of various MRI slices. Early detection of tumors helps in disease treatment and increases the chances of survival for many patients [6]. However, the radiologists are often faced with vast amounts of MRI data and multiple complex tumors. This leads to a high risk of error and a long treatment process, especially when small slices are affected. As a result, brain tumor diagnosis and discrimination between various brain tumor types are complex tasks.

**Figure 2:**
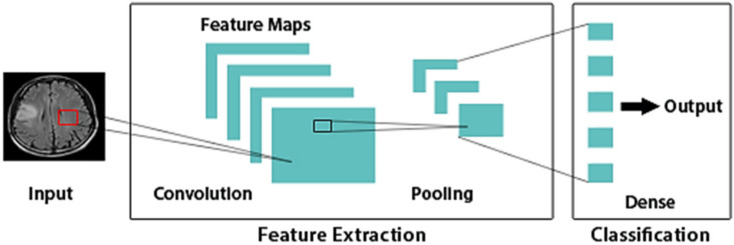
Simple CNN architecture. Dense: Fully connected layer.

CNN is the most recent and widely used Deep Learning method within medical image analysis. In general, CNNs are typically designed to deal with raw images and used to minimize data pre-processing steps [7,8,9]. CNN’s design is based on the brain’s structure. The nodes in CNN operate in the same way as neurons in the brain do in processing and transmitting messages throughout the body: they accept inputs, analyze them, and then deliver the results as an output. The image is provided into the algorithm as input. The input layer takes image pixels as information in the array form. It is possible for CNN to have many hidden layers, each of which executes feature extraction from an input image by performing computations. Examples of these layers include convolution, pooling, and dense layers. An input image’s features are initially extracted using convolution. In order to minimize the size of feature maps, pooling is utilized. Object classification and identification are performed by the dense layer. CNNs, similar to artificial neural networks, are based on biological concepts. Their design is motivated by the brain’s visual cortex, which is composed of alternating layers of complex and simple cells. Representation of a simple CNN architecture is depicted in Figure 2. Using CNN, the traditional handcrafted features are no longer necessary since it automatically learns the features that are important for making correct predictions on its own. Nevertheless, there is a limitation in exploring hyperparameter space. As evidenced by the previous models, they failed to attain an accuracy comparable to near-perfect, which is unpalatable in a clinical setting. The proposal of this paper is then to use Bayesian Optimization, which is a reasoning-based method, to select the optimal hyperparameters in the shortest amount of time possible.

This paper makes the following major contributions:

- A robust CNN architecture is presented for automated classification of the most common brain tumor types;
- The optimal hyperparameters are automatically selected by adopting Bayesian Optimization;
- Extensive performance evaluation is reported by comparing the optimized model against pre-trained VGG16, VGG19, ResNet50, InceptionV3, and DenseNet201 models;
- The optimized model has achieved higher accuracy in brain MRI images compared to other state-of-the-art methods.

The remaining sections of the paper are described as follows: Section 2. Discusses the related works. Section 3. Presents the proposed methodology in detail. Section 4. Demonstrates the experimental results and the comparison of existing methods. Lastly, Section 5. Concludes the study.

## 2. Related Works

Numerous works have already been completed on the classification of brain MRI images by CNN for its superior accuracy. Cheng et al. [10] developed a method to improve brain tumor classification performance by augmenting tumor region of interest (ROI) through image dilation followed by splitting into subregions. For features extraction, they used Gray Level Co-occurrence Matrix (GLCM), intensity histogram, and Bag of Words] (BOW). This method had 91.28% classification accuracy. Paul et al. [11] in their work proposed a CNN model that has two convolutional layers, two max-pooling layers followed by two fully connected layers. As the used dataset contained three different planes (axial, coronal, and sagittal), they selected only axial images to prevent confusion in the model. They achieved an accuracy of 91.43% on their experimental analysis. Muhammad Sajjad et al. [12] introduced a system that classifies multi-grade brain tumors. The system includes three phases, which are: the segmentation of the tumor region through a CNN model, the augmentation of the segmented data to increase the number of images, and fine-tuning the pre-trained VGG19 for multi-grade brain tumor classification. This method obtained 96.56% classification accuracy. Ahmet Çinar et al. [13] used the pre-trained ResNet50 model as a base. Then, they removed its last five layers and added 10 new ones in its place. The updated ResNet50 model demonstrated robust results by gaining 97.2% classification accuracy. Sunanda Das et al. [14] presented a system that includes two key steps. The first is preprocessing the images using Gaussian filter along with Histogram equalization, and the second is to classify the preprocessed images using the CNN model. This system attained an accuracy of 94.39%, a precision of 93.33%, and a recall of 93%. Abiwinada et al. [15] trained a simple CNN architecture to classify the three most prevalent brain tumor types, i.e., Meningioma, Glioma, and Pituitary without prior region-based pre-processing steps. They identified an optimal CNN model containing two convolutional layers, activation function (ReLU), max-pooling, and one fully connected layer. Their classification model reached 98.51% training accuracy and 84.18% validation accuracy. Khwaldeh, saed et al. [16] proposed a framework to classify brain MRI scans into healthy and unhealthy, along with a grading system to categorize the unhealthy brain MRI scans into low and high grades, through the modification of the Alex-Net CNN model. This framework gave an accuracy of 91%. Hossam H Sultan et al. [17] introduced multi-classification of brain tumor images by deep neural network based on two available datasets. An accuracy of 96.13% is obtained for the classification of the tumors into Meningioma, Glioma, and Pituitary. While an accuracy of 98.70% is achieved for the classification of the three grades of Glioma (Grade II, Grade III, and Grade IV).

Bringing Deep Learning into the medical healthcare field is hampered by the lack of labeled data. As the recent growth of Deep Learning implementations in other fields has proven, the larger the data, the better accuracy the results will be. Deep Learning is used for data segmentation and augmentation in the above literature. The majority of literature discusses the effectiveness of using Transfer Learning to perform classification. VGG19 and ResNet50 are the deep pre-trained models that are often used in the mentioned literature which are pre-trained on a huge dataset (i.e., ImageNet). If the dataset is small, we apply fine-tuning to minimize parameters. The downside of Transfer Learning is the possibility of negative transfer. The initial and target problems must be similar for the first training round to be important. Otherwise, Transfer Learning will be ineffective [18]. One other drawback of Transfer Learning is that the size of the input image is fixed. The images must be adjusted according to the pre-trained model’s input size. It is also worth noting that according to the mentioned research works, Bayesian Optimization technique has not yet been applied to automate the selection of optimal hyperparameters in the domain of brain tumor classification. Therefore, this paper aims to explore the implementation of Bayesian Optimization-based CNN on classifying different brain tumor types, thereby improving the performance and proving the efficiency of training an optimal CNN model from scratch over Transfer Learning.

## 3. Materials and Methods

**Figure 3:**
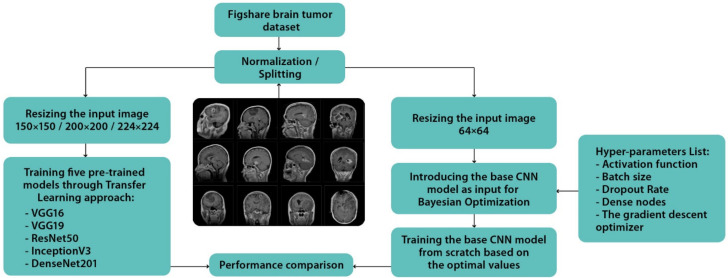
The proposed approach.

Figure 3 summarizes the proposed approach, which is further explored in the subsections that follow.

### 3.1. Dataset and Preprocessing

**Figure 4:**
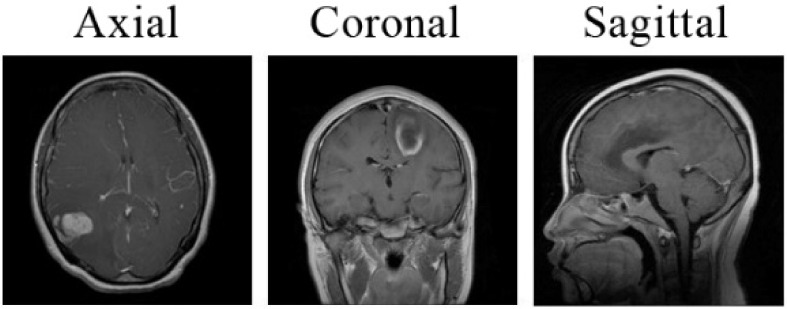
Representation of the three different planes of the MRI images.

The necessary data for this method are collected from the Figshare brain tumor dataset. This dataset was obtained from General Hospital and Nan fang Hospital, Tianjin Medical University, China and proposed online by Cheng, Jun, et al. [19]. It is available on the Figshare website (https://figshare.com/articles/dataset/brain_tumor_dataset/1512427) (accessed on 2 January 2022) for anyone to download in MATLAB “. mat” format. It contains a sum of 3064 T-1 weighted contrast-enhanced MRI images of three different varieties of brain tumor (Meningioma, Glioma, and Pituitary). The number of images in each class is listed in Table 1. The MRI scans were provided in three planes: axial (994), coronal (1045), and sagittal (1025), as displayed in Figure 4. In order to maintain variety in the images, the dataset is randomly divided into two parts. In total, 90% of the dataset was reserved for training and 10% for the validation data. Data normalization is used after converting the image to an array of pixels in order to rescale the image’s pixels to the range of 0–1. All MRI images were originally provided in 512 × 512 size. These images represent the input layer of the base CNN architecture. Inspired by [15], we resized them to 64 × 64 pixels. This reduction is performed to speed up the training process and reduce the memory requirement. Note that this resizing operation retains all of the information included in each MRI image, implying that no cropping or segmentation is undertaken here.

### 3.2. The Base CNN Network Architecture

The use of CNNs in machine vision is quite successful [20]. From the conceptual perspective, CNN is similar to a multilayer perceptron (MLP). In MLP, each neuron has its activation function, which connects the weighted inputs to each neuron’s output. When there is more than one hidden layer, the MLP becomes a deep MLP. A CNN is similar to an MLP, but it has an exceptional structure. This exceptional structure in the architecture allows it to be translation and rotation invariant at the same time [21]. The overall structure of the CNN architecture contains an input layer, convolutional layers, pooling layers, one or more fully connected layers, a classification layer, and finally, an output layer [22,23]. The convolutional layer is the main component of CNN. It performs what is known as a “convolution operation” which is a process that involves applying a filter to an input that produces an activation. By using convolutional layers, characteristics in the image can be extracted, including edges, textures, and objects. The feature maps are created as a result of updating the filter weights during the training process [24]. The pooling layer is used to reduces the dimension of the last layer and comes in two types: max-pooling and average-pooling. It can be regarded as a feature extractor when the convolution and pooling layers are combined [25]. The classification phase is carried out using the fully connected layers [26].

In this paper, a base CNN architecture is first created, before hyperparameters optimization can be performed. This proposed architecture contains an input, five main blocks (five convolutional and five max-pooling layers), and a classification block (two fully connected and one dropout layer). This topology was found to be the best fit for this classification task through experiment. Starting from the input layer which holds the MRI images from the preceding pre-processing stage passing through the first main block, this block has a convolutional layer that applies 32 2-D convolutional filters of size 3 × 3 to all the images with zero-padding, so the input image becomes fully covered by the filter. Then, a max-pooling layer of 2 × 2 size is used to gain robustness on feature extraction with a stride of two pixels. The other four main blocks differ from the first block only in the number of filters of the convolutional layer. The second, third, fourth, and fifth main blocks use 64, 64, 128, and 256 2-D convolutional filters, respectively. The classification block has two fully connected layers and a dropout layer. Dropout prevents overfitting by ignoring random neurons during training [27]. The last fully connected layer outputs the values for three tumor classes (1. Meningioma, 2. Glioma, and 3. Pituitary tumor) using the Softmax activation function [28]. Equation (1) describes the mathematical formula of Softmax activation.

(1) $$\sigma {\left(z\right)}_{i}=\frac{e^{z_{i}}}{\sum_{j=1}^{K}e^{z_{j}}}$$

For i = 1, 2 …, K and z = $(z_{1},\;z_{2}\;\ldots ,z_{K})\in \;{\mathbb{R}}^{K}$. Each class’ probability score is calculated by this function. Note that this stage did not specify.

- The activation function for the convolutional layers and the first fully connected layer;
- The dropout rate;
- The number of nodes in the first fully connected layer.

**Figure 5:**
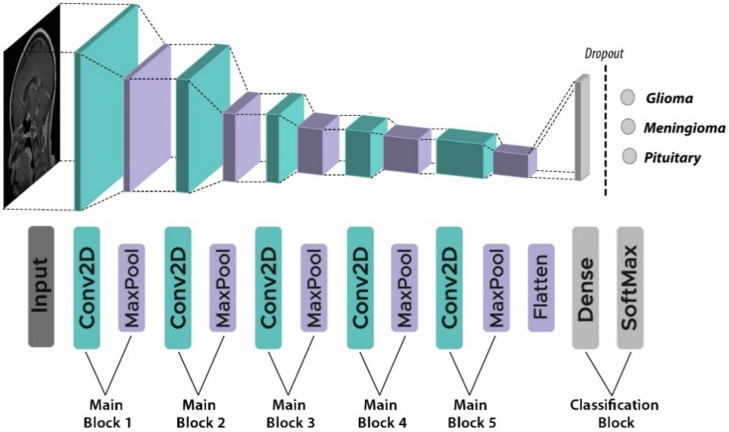
The architecture of the base model (five convolutional layers, five max-pooling layers, two fully connected layers and one dropout layer).

They are among the hyperparameters that will be chosen later for optimization. The architecture of the base model is presented in Figure 5. This proposed architecture is a slightly modified version of the VGG16 concept [29], with the number of convolutional layers lowered from 13 to 5 and the number of dense layers reduced from four to two. In addition, we added a dropout layer between the dense layers and kept the number of max-pooling layers the same as the VGG16. In other words, we lowered VGG16’s complexity.

### 3.3. Hyperparameters Optimization

The objective of this study is to optimize the base model hyperparameters for classifying various types of brain tumors using MRI images. There are two main categories of model parameters in Machine Learning: Parameters, which are learned from data and they cannot be manually altered by the user such as the weights of neural network. Hyperparameters, whose values cannot be learned from data and they can be set before the training operation by the user such as the number of dense nodes or dropout value in the model.

Hyperparameters optimization aims to maximize the performance of a given Machine Learning algorithm by selecting the most suitable hyperparameters [30]. Based on Equation (2), where f denotes the performance, x is said to be some hyperparameter setting, and the optimum choice is $x_{opt}$.

(2) $$x_{opt}=\underset{x\in X}{\mathrm{argmax}}f\left(x\right)$$

Various methods are available to achieve this. The most common one is Grid Search [31], which is a process for searching exhaustively through a subset of a hyperparameter space for a targeted algorithm. As more hyperparameters are added, the number of parameters-combinations will also increase exponentially. As a result, the process will be extremely time-consuming. Another way to find useful hyperparameters is Random Search [31]. Unlike Grid Search, Random Search experiments with various combinations of parameters entirely at random. This produces high variance when computing. These two methods are unable to learn anything during the tuning process from the evaluated hyperparameter sets.

**Figure 6:**
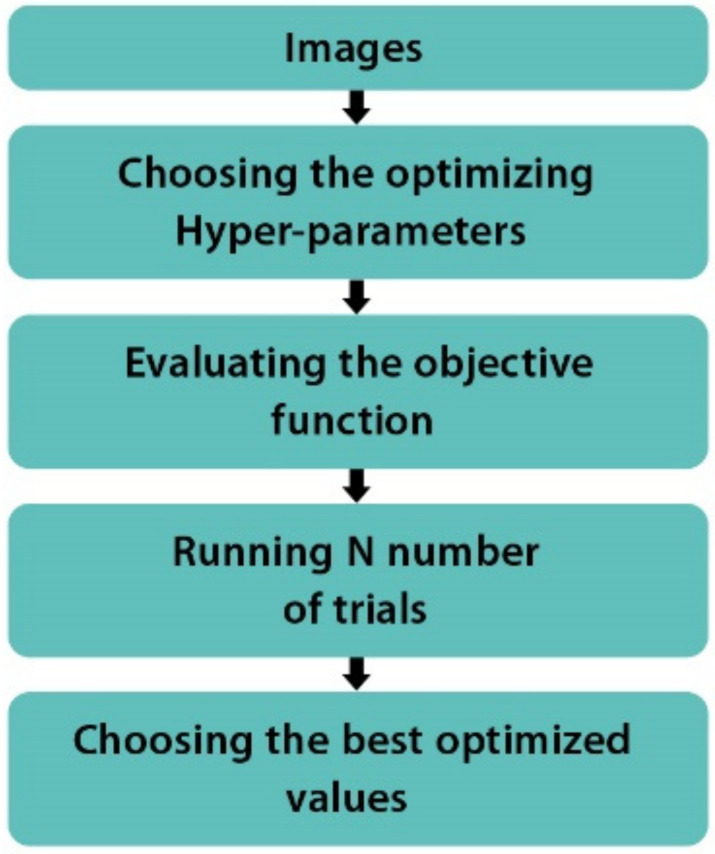
The flow of Bayesian Optimization.

In this study, a clever technique for obtaining optimal hyperparameters is used, which is known as Bayesian Optimization [32]. Among the reasons for selecting Bayesian Optimization are that previous studies have proven its superiority to Grid Search [33] and that unlike Grid Search, Bayesian Optimization is capable of efficiently finding optimal hyperparameters with fewer iterations [34]. Bayesian Optimization employs a surrogate model that is fitted to the real model’s observations. In our case, an observation is a complete training of the base CNN model with hyperparameters chosen specifically for that observation. A set of hyperparameters is selected for each iteration, and an observation is then made. The validation accuracy is used for the evaluation of the observation. The hyperparameter set is selected using an acquisition function that balances the choice between exploring the entire search space and exploiting well-performing areas of the search space. The acquisition function Expected Improvement [35] is used in this study to carry out Bayesian Optimization experiment. Figure 6 shows the flow of Bayesian Optimization.

Bayesian Optimization is implemented using the Scikit-optimize 0.8.1 (also known as Skopt) [36] Python package. Skopt allows various parameters to affect the optimization performance. We can pass a function for optimization, specify the number of optimization iterations, and display the graph of the optimization process. Furthermore, we can provide a listing of hyperparameters along with their search dimensions. In addition, we can increase the number and bounds of hyperparameters.

The hyperparameters selected to be optimized are: the activation function, the batch size, the dropout rate, the number of dense nodes, and the gradient descent optimizing function. These hyperparameters are among the most important for the accuracy and the success of this multi-classification task. The valid search ranges for each of these hyperparameters are specified. The activation functions specified are “ReLU, ELU, Sigmoid, SELU, and Tanh”, the batch size is between 1 and 128, the dropout rate has a lower bound of 0.1 and an upper bound of 0.5, the low and high of the number of dense nodes is 32 to 1024 and the optimizers specified are Adam (adaptive moment estimation), Nadam (Adam with Nesterov momentum), AdaMax (an Adam variant that uses the infinity norm), RMSProp (root means square propagation), and SGD (stochastic gradient descent). The hyperparameters considered in the Bayesian Optimization experiment are given in Table 2 along with the search dimension for each hyperparameter.

### 3.4. Transfer Learning

In Deep Learning, sometimes we use a pre-trained network instead of training a model from scratch. A pre-trained network is a saved model which has been previously modeled on a dataset such as ImageNet [37]. ImageNet comprises 1000 categories of ordinary objects such as cats, books, and houses. In spite of the differences from images in the medical domain, these images share the characteristic of being natural with lights, contrasts, and colors. These shared characteristics are the ones that should be preserved while fine-tuning to the Figshare brain tumor dataset.

In this experiment, pre-trained VGG16, VGG19, ResNet50, InceptionV3, and DenseNet201 models are fine-tuned according to the target data to prevent overfitting. A comparison is then made between the performance of each model and that of the optimized model. In all five pre-trained models, ReLU, dense, and dropout layers are used. The Softmax function is selected as a classification layer. The input images for VGG16, VGG19, and InceptionV3 are 224 × 224 pixels, 224 × 224 pixels, and 150 × 150 pixels, respectively. The other two CNNs had inputs of 200 × 200 pixels. The pre-trained models’ input sizes varied depending on the network’s size requirements, and this was taken into account while setting the input sizes. For instance, InceptionV3’s input shape must be between 299 × 299 pixels and 75 × 75 pixels. Each of these pre-trained models is trained using a batch size of 32. Adam [38] is chosen for optimization with a learning rate of 5 × 10^−5^. The dropout rate is selected as 0.5 to regularize the deep models. The parameter values used for each pre-trained model are provided in Table 3. The subsections that follow describe each pre-trained model in greater detail.

#### 3.4.1. VGG16 and VGG19

**Figure 7:**
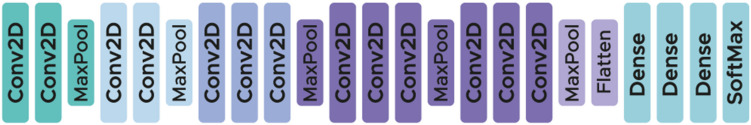
The structure of VGG16.

VGG16 is a CNN model introduced by Andrew Zisserman and Karen Simonyan in 2014. This model achieves 92.7% test accuracy in the ImageNet dataset, which contains 14 million images. The network is comprised of 16 layers in total including multiple layers of kernels, resulting in a deeper neural network. This makes it capable of understanding and recognizing more complex patterns and features. In VGG16, we have convolutional layers, average-pooling layers, and dense layers. An RGB image of 224 × 224 pixels is used as an input for the first convolution layer. The neural network has an initial width of 64 and its width doubles after every pooling layer. The first two dense layers have 256 channels each, while two channels are present in the third layer. ReLU is used on the two first fully connected layers, and Softmax is used on the final layer. After each 256-channel fully connected layer, dropout was applied. The network’s learning rate is 0.0001. The cost function has been the categorical cross entropy with Adam optimization [29]. The structure of VGG16 is demonstrated in Figure 7.

**Figure 8:**
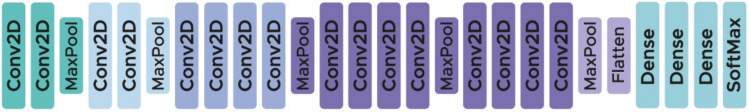
The structure of VGG19.

In VGG19—a variant of VGG16—there are 19 layers convolutional neural network including 16 convolution layers, five max-pooling layers, three fully connected layers, and a Softmax layer. The basic architecture of this model is the same as that of VGG16. VGG19 only differs in that it uses two fully connected layers of 256 each and two channels, and also the reduction of the learning rate to 0.00001 [39]. The structure of VGG19 is illustrated in Figure 8.

#### 3.4.2. ResNet50

**Figure 9:**
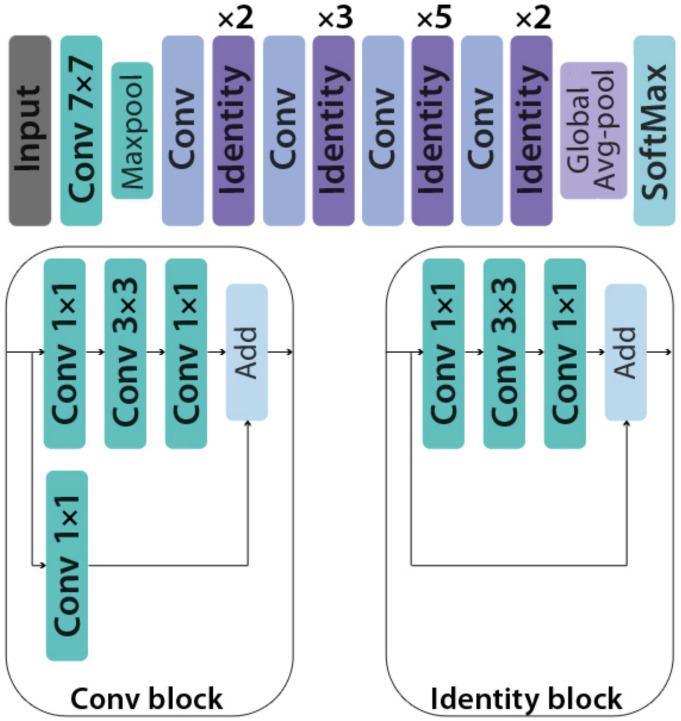
The structure of ResNet50 model.

ResNet50 is a CNN model from ResNet (Residual Networks) family containing twenty-six million parameters. This 50-layer model was introduced by Microsoft in 2015, and it comprises the identity and conv blocks [40]. The 3 × 3 filters are used in the network’s convolutional layers and direct down sampling is achieved by the convolutional layers having a stride of two. The final layer within the network is a fully connected layer with 256 dense nodes and a ReLU activation function. The structure of ResNet50 is presented in Figure 9.

#### 3.4.3. InceptionV3

**Figure 10:**
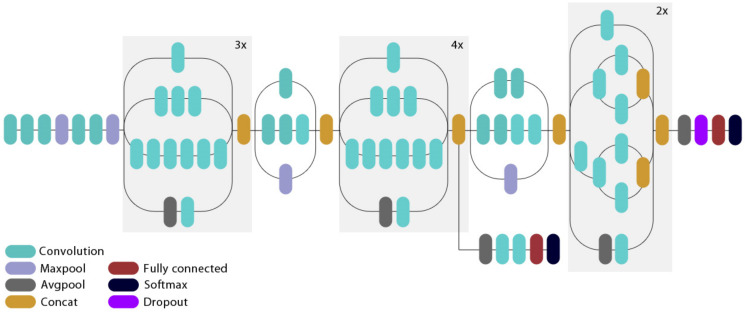
The structure of InceptionV3 model.

The inception model (GoogleNet) was presented by Google in 2014 [41]. InceptionV3 is a part of the Inception family, which includes 42 layers with 24 million parameters. It introduced changes to the Inception module in order to increase ImageNet classification accuracy. As a result of the additional factorization, the number of parameters was reduced without a reduction in network efficiency. The network was among the first to apply batch normalization to the layers. The structure of InceptionV3 is displayed in Figure 10.

#### 3.4.4. DenseNet201

**Figure 11:**
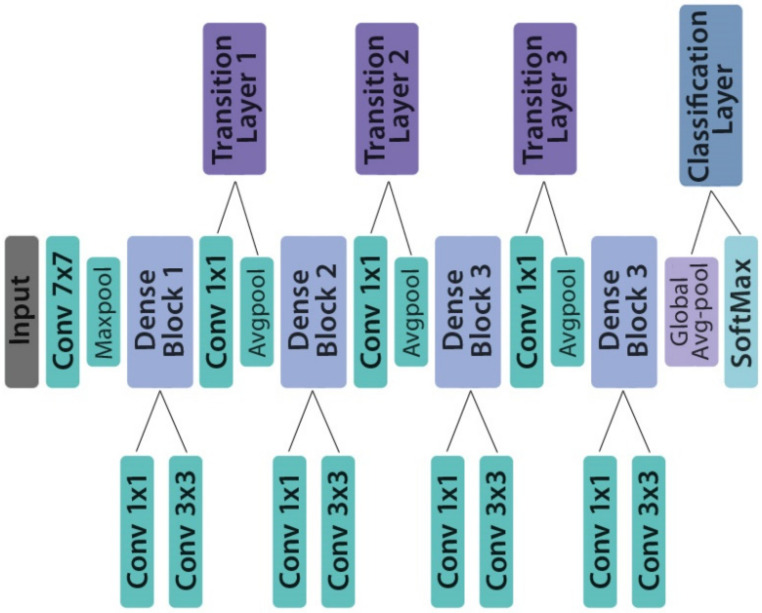
The structure of DenseNet201 model.

DenseNet201 architecture is one of the DenseNet group of architectures designed to perform image classification [42]. It was the winning model in the 2015 ImageNet challenge. Layers in DenseNet can access directly the original input image and the Loss function’s gradients. Due to this, the computation cost is significantly reduced, which makes DenseNet an excellent choice for image classification tasks [43]. Pre-trained weights from the ImageNet database were loaded into the network. The structure of DenseNet201 is depicted in Figure 11.

### 3.5. Evaluation Metrics

The performance of the proposed approach is analyzed using accuracy, precision, recall, f1-score, loss, and confusion matrix [44,45,46,47,48]. Accuracy refers to the degree of closeness between an estimated value and its original value in the classification process. Mathematically it is represented as:

(3) $$\mathrm{Accuracy}=\frac{TP+TN}{TP+TN+FP+FN}$$

where TP, TN, FP, and FN indicate the number of classified cases of true positives, true negatives, false positives, and false negatives, respectively.

Precision is defined as the positive predictive rate (PPR) and it is represented as:

(4) $$\mathrm{Precision}=\frac{TP}{TP+FP}$$

Recall, also called sensitivity, describes how well the classifier classifies the correct tumor types and it is represented as:

(5) $$\mathrm{Recall}=\frac{TP}{TP+FN}$$

F1-score represents how well the classification has performed in terms of recall and precision and it is represented as:

(6) $$F1-\mathrm{score}=2\times \frac{Precision\times Recall}{Precision+Recall}$$

Loss is defined as the cost of inaccurate predictions in classification task. The Categorical Crossentropy Loss Function is employed for loss calculation. It computes the difference between target values and predicted values. It can be represented using the following mathematical formula:

(7) $$\mathrm{Loss}=-\frac{1}{n}\overset{n}{\underset{j=1}{\sum }}\overset{K}{\underset{k=1}{\sum }}Z_{k}^{j}\log({\hat{Z}}_{k}^{j})(1-Z_{k}^{j})\log(1-{\hat{Z}}_{k}^{j})$$

where k denotes class, K represents the total number of classes, j is sample number, ${\hat{Z}}_{k}$ demonstrates the predicted value, $Z_{k}$ denotes the ground truth value, and n is the sample number in a batch.

The confusion matrix displays how confused the classification model is for each class by describing the relationship between the predicted results and the expected values. There is growing evidence that the confusion matrix is useful for model validation due to its robust categorization [48].

## 4. Results

This section describes the experimental results that demonstrate the effectiveness of the proposed method.

### 4.1. Experiment Setup

Training and execution of all models were performed using Google Colaboratory [49] (also known as Colab) (https://colab.research.google.com) (accessed on 13 January 2022). This cloud service is based on Jupyter Notebooks and it provides a virtual GPU powered by NVIDIA Tesla K80 with 12 GB RAM. The Keras [50] library was adopted along with TensorFlow [51] to build the Deep Learning architectures.

### 4.2. Results of Bayesian Optimization Experiment

In this experiment, A total of 40 iterations have been completed, which can be expanded to 80–100 for more improvement. For each iteration, the maximum number of epochs was 30. In total, the 40 iterations were carried out in 1 h, 34 min, and 28 s. The Keras callback class was used in each iteration to stop training when the validation accuracy reaches or surpasses 98%, this helps to reduce overfitting.

**Figure 12:**
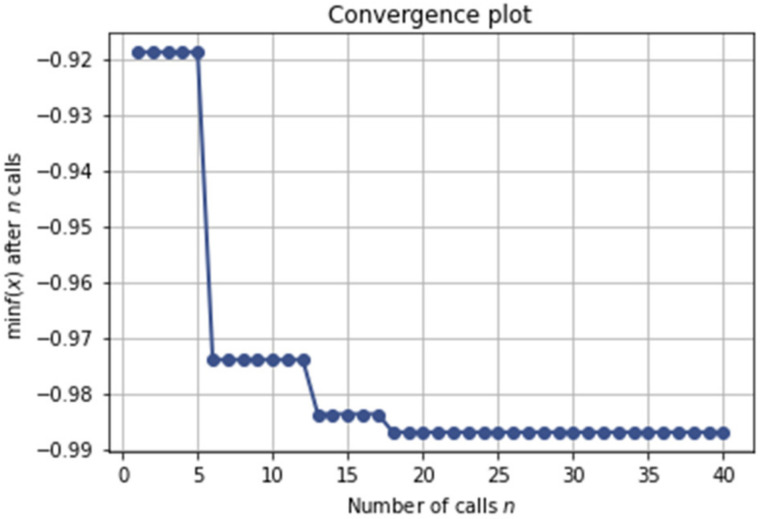
The progress of the hyperparameter optimization.

Figure 12 shows the convergence trace at different iterations during the optimization process including the optimal point. It took only a few hyperparameters trials to find significant improvements. After 18 iterations, we can see the minimum in the function value has already converged and the accuracy score does not improve after that.

**Figure 13:**
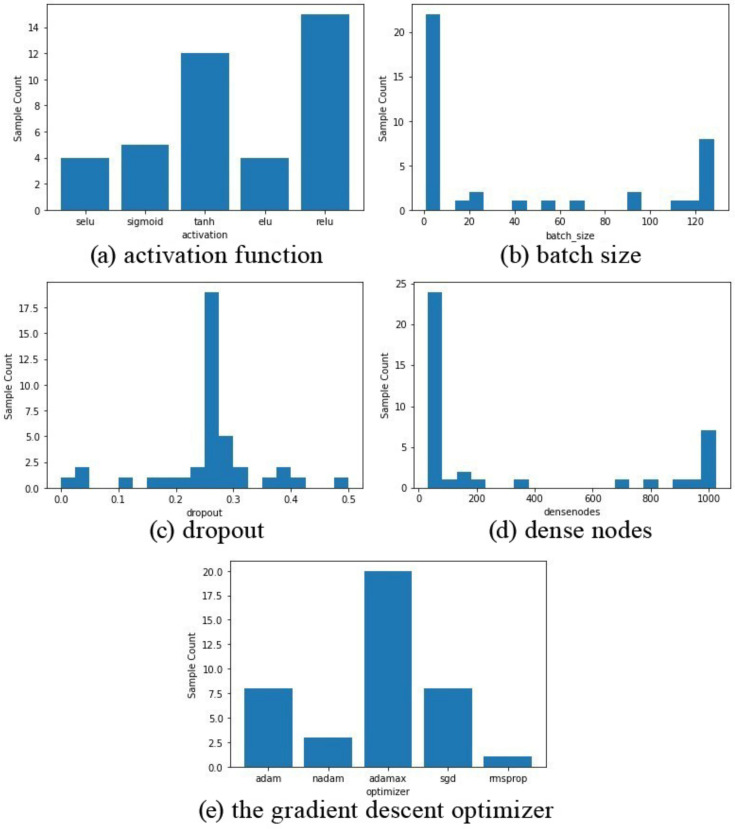
Count of the hyperparameters selected. (**a**) activation function; (**b**) batch size; (**c**) dropout; (**d**) dense nodes; (**e**) the gradient descent optimizer.

The histograms of different values selected for various epochs are indicated in Figure 13. As shown in Figure 13a, the ReLU activation function has been selected nearly 15 times, while Tanh was in second place with 12 sample counts. In Figure 13b, the lowest value for the batch size dimension was chosen more than 23 times. It can be seen in the histogram shown in Figure 13c, 0.25 range of dropout have the highest selection count up to 17 times. The histogram in Figure 13d shows that a majority of the time, the number of dense nodes is less than 50. Figure 13e represents the choice of the gradient descent optimizer, as can be seen, Adamax was the optimizer selected almost 20 times.

**Figure 14:**
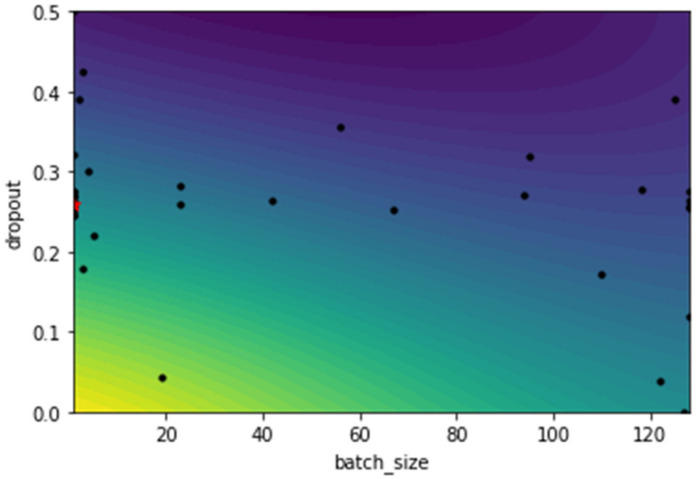
The 2D-landscape plot of dropout and batch size.

Bayesian Optimization constructs a surrogate model of search dimension and starts searching within that instead of the real dimension. Thus, accelerating the search process. A 2D-landscape plot of two optimized hyperparameters (batch size and dropout rate) is displayed in Figure 14. According to the plot, blue is the worst region and yellow is the best. The sampling location of the optimizer is indicated by black dots whereas the red-star on the left presents the best hyperparameter value discovered. As we can see, different areas have been investigated, especially those where dropout rates are between 0.2 to 0.3. The optimal values for dropout and batch size were 0.25 and 1, respectively, and this is what the red star pointed to.

There were various models that exceeded 98% validation accuracy. Thus, one of them had to be chosen. The top five most accurate models are displayed in Table 4. It is observed that the highest accuracy obtained is 98.70%. The two top models (trial number 17 and 38) both achieved the same accuracy. However, the first one took less training time and fewer epochs to surpasses 98% accuracy. Hence, it was deemed to be the optimal model. Further, it is evident that the optimizer and the activation function affect how long it takes to reach the target accuracy. When Tanh activation was applied with SGD optimization, the full training time was three times shorter than for Nadam with ReLU. A summary of the optimal model is provided in Table 5.

Having found a decent set of hyperparameters by hand tuning is helpful in starting the search. However, to prove the usefulness of hyperparameter optimization, the values for the first iteration were chosen at random (Selu activation functions, a batch size of four, a dropout rate of 30%, 32 nodes for the dense layer, and Adam as optimizer). Table 6 compares the performance of the base model before optimization and after optimization. Before optimization, the validation accuracy is 91.88% and the training time is 117 s, whereas after optimization, the validation accuracy is improved, which is 98.70% and the training time is decreased to 99 s. An enhanced performance was evident in the scratched model after Bayesian Optimization. The term “scratched model” refers to a model that is built from scratch on a dataset, unlike Transfer Learning, where a model created for one task is repurposed for another. The scratched model in our case refers to the Base CNN model.

Table 7 demonstrates the effect of training-validation data size on the performance of the optimized model. A total of five split styles (90–10%, 80–20%, 70–30%, 60–40%, and 50–50%) are used for training-validation data because data size is an important factor that can impact CNN performance. It is noticed that the best accuracy is achieved when 90% of the data samples are used in training. Additionally, we can see a slight reduction in classification performance even with 50% of the training data.

### 4.3. Comparison with Five Deep Pre-Trained CNNs

In this experiment, five deep pre-trained CNNs were trained on the same CE-MRI dataset using the Transfer Learning approach. The dataset of 3064 images was split in the same way as in the previous step (90% of images were used for training and 10% for validation). The training was conducted for 11 epochs in order to maintain an equitable comparison. The model-checkpoint Keras callback is executed at the end of each epoch to save whenever the validation accuracy improved. Each class (Glioma, Meningioma, Pituitary) was evaluated using the following metrics: precision, recall, f1-score, accuracy, and loss of the model.

A comparison of the optimized model with five pre-trained models can be found in Table 8. It can be noted that the optimized model, VGG16, and VGG19 achieved the best classification accuracy of 98.70%, 97.08%, and 96.43%, respectively. DenseNet201 achieved a moderate accuracy of 93.51%. The accuracy of the other models did not exceed 93%, with ResNet50 having the lowest result with 89.29%. Regarding the optimized model, a precision of 97% is observed for the Meningioma class whereas the five pre-trained models did not surpass 92%. Furthermore, the aforementioned model would accurately identify “Glioma and Pituitary” 99% of the time. With an average of 98.33% precision, 98.66% recall, and 98.66% f1-score, the optimized model comes out the best among the other models.

**Figure 15:**
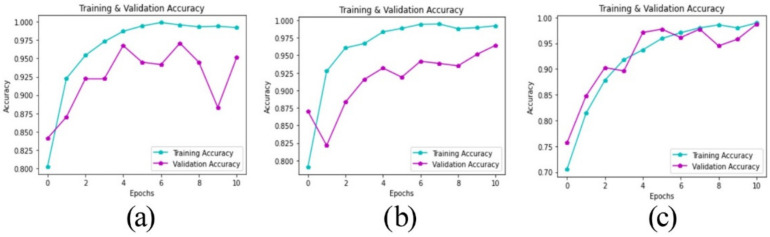
Accuracy plots (training and validation). (**a**) VGG16. (**b**) VGG19. (**c**) The optimized model.

Figure 15 presents the classification accuracy of the optimized model along with the two best pre-trained models (VGG16 and VGG19). The *x*-axis represents the number of training epochs, while the y-axis reflects the accuracy. The optimized model demonstrates a smooth training process during which the accuracy gradually increases until the end. Moreover, it is noticeable that the training and validation accuracy adhere to one another in most cases indicating that the optimized model does not overfit. On the other hand, the training and validation of VGG16 and VGG19 deviate much from one another most of the time, which indicates overfitting. Particularly in VGG16, the validation accuracy fell dramatically between eight to nine epochs.

**Figure 16:**
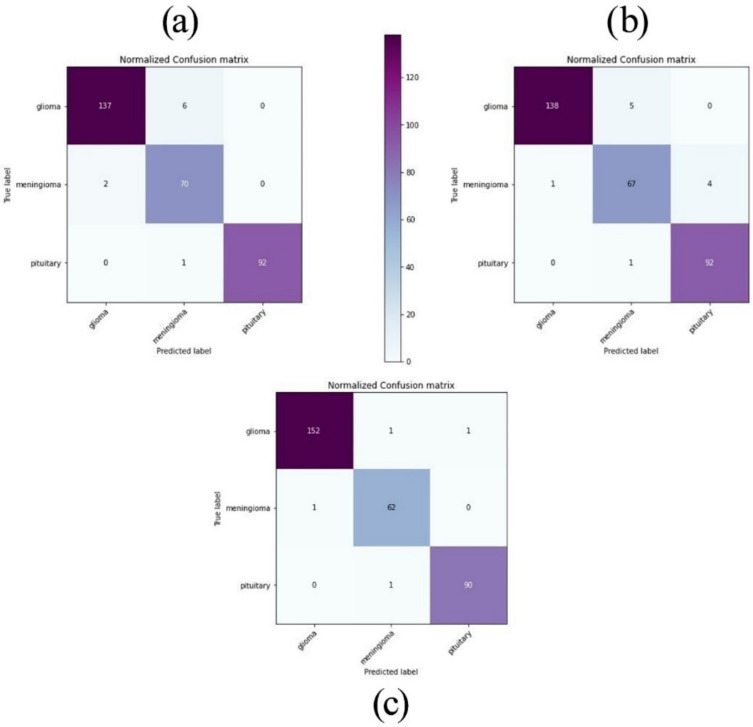
Confusion matrices of the optimized model, VGG16, and VGG19. (**a**) VGG16. (**b**) VGG19. (**c**) The optimized model.

The confusion matrices shown in Figure 16 define how many images in the validation set were correctly classified according to the optimized model, VGG16 and VGG19. The rows of the matrix represent the expected values (ground truth) while each column corresponds to the predicted results (system output). It can be observed that the number of miss-classified images with the optimized model was significantly lower as compared to VGG16 and VGG19. Besides that, the two pre-trained models are sometimes confused with Meningioma since this class had a lower number of images than Glioma and Pituitary.

**Figure 17:**
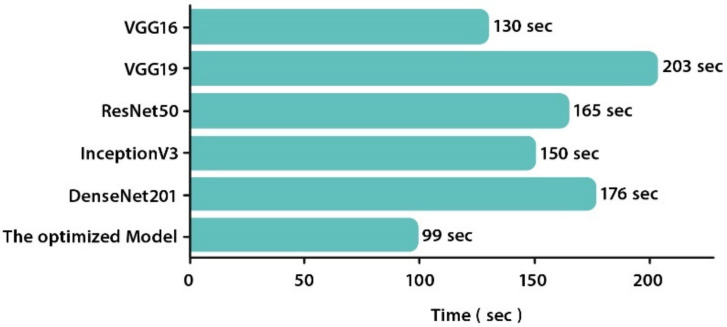
Execution time of all CNNs on GPU.

When the processing time was regarded as a benchmark criterion, VGG19 exhibits the longest execution time followed by DenseNet201 and ResNet50. It makes sense since these models have the greatest number of parameters. The optimized model presented the lowest execution time (99 s). It converged 1.3, 2, 1.6, 1.5, and 1.7 times faster than VGG16, VGG19, ResNet50, InceptionV3, and DenseNet201, respectively, and is thus proven to be the most efficient model for this brain tumor multi-classification task. Figure 17 illustrates the execution time of each CNN (in seconds).

### 4.4. Comparison with State-of-the-Art Methods

In order to provide a proper evaluation of performance, the optimized model was compared to previous studies that used the same CE-MRI dataset. The classification results of the five compared methods were taken from the corresponding original papers, as stated in Section 2. Table 9 displays the comparison results in terms of accuracy metric. The comparison shows that the optimized model is the more accurate in classifying brain tumors in MRI images. The deep neural network of Hossam H Sultan et al. [17] also performs well with an accuracy of 96.13%. In summary, we established that the employment of the Bayesian Optimization method yields impressive results even for a small training dataset.

### 4.5. Evaluation on Unseen Data

In order to evaluate the capacity of our optimized model to accurately classify MRI scans into glioma, meningioma, and pituitary on completely unseen data. We used another publicly available dataset from the Github website [52]. It contained 2870 brain MRI images (826 glioma tumors, 822 meningioma tumors, 395 no tumor, and 827 pituitary tumors). This dataset has already been divided into train and test sets. Our study used only the test set of MRI images. Moreover, we omitted the “no tumor” images because they weren’t considered in this study.

**Figure 18:**
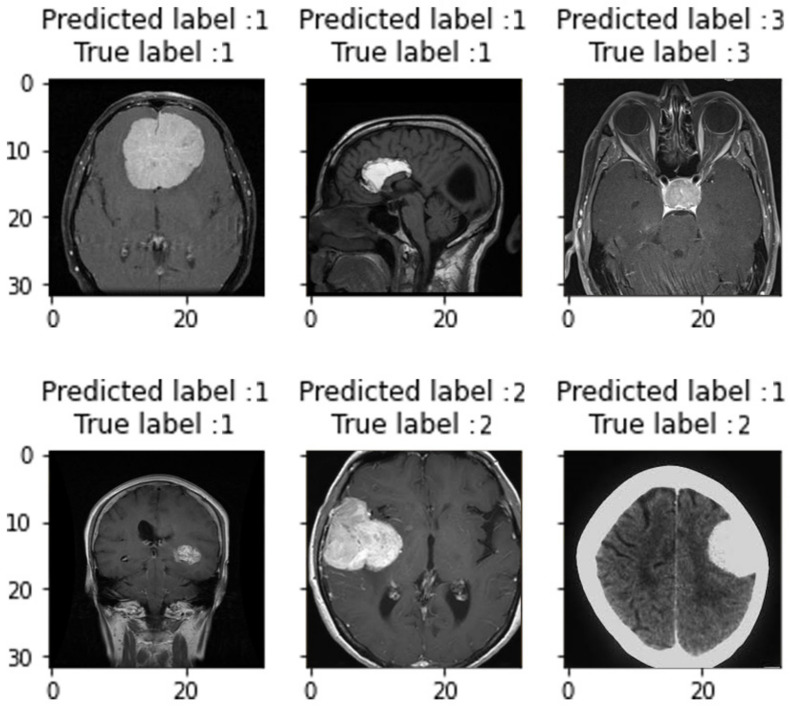
Samples of predicted labels against true labels on unseen data.

Our optimized model attained a Precision of 97.63%, a Recall of 97.59%, an F1-score of 97.01%, and an accuracy of 97.52%. Clearly, these values are “very good” in terms of medical diagnosis, and can be further improved if more data are available. Our model’s performance on unseen data is still better than the performance of recent studies listed earlier in Table 4. In terms of accuracy, we exceeded the second-best technique (Hossam H Sultan et al. [17]) by 1.39%. Figure 18 depicted samples of predicted labels against true labels on this unseen data. Note that labels 1, 2, and 3 represent Glioma, Meningioma, and Pituitary tumors, respectively. It is observed that our model can predict the real labels in the majority of cases, reflecting the accuracy reached. Only one image from these six samples was mistakenly recognized; this is due to the low quality of the MRI scan in that case; as can be seen, the tumor is not clearly evident. There is also a white wide stripe surrounds the brain, which confused our model.

This model has yet to be confirmed in real-world clinical practice, implying that we are still in the theoretical research phase. In the future, we plan to speak with radiologists to see how such a model can fit in a real-world clinical scenario.

## 5. Conclusions

Brain tumor classification is among the most crucial aspects of the medical field. Building an efficient CNN is not an easy task. For this reason, it has become essential to use optimization methods to set CNN hyperparameters. This paper proposed a new approach that classifies among three common brain tumor classes (Glioma, Meningioma, and Pituitary). The optimal hyperparameters values of the model are selected using Bayesian Optimization technique. On the other hand, five pre-trained CNNs were finetuned and trained on the same dataset. Research findings indicated that the optimized model gave the best classification performance with 98.70% accuracy followed by VGG16 with 97.08% accuracy whereas ResNet50 attained the lowest results with 89.29% accuracy. It is concluded that automating hyperparameter optimization is effective in increasing the performance of a scratched CNN model. Training and validation are conducted using MRI scans in axial, coronal, and sagittal planes. The model presented in this paper can be extended to classify other diseases effectively.

Future research can address some of the limitations of this paper. An extensive analysis requires a lot more patient data, particularly for the meningioma class, which had the lowest number of images of all three classes studied. Moreover, further research should focus on tuning more hyperparameters such as the number of convolutional layers, the number of filters on each convolutional layer, the kernel size, and the number of fully connected layers. Besides, further improving the proposed model can be achieved by increasing the search dimensions for the hyperparameters. Finally, by adding normal brain CE-MRI images to the dataset, more differentiation can be provided for tumor classification.

## Figures and Tables

**Table 1:** The dataset used for the proposed approach.

Tumor Class	Number of Images
Meningioma	708
Glioma	1426
Pituitary	930
Total	3064

**Table 2:** Hyperparameters setting.

Hyperparameter	Range to Probe
Activation function	ReLU—ELU—Sigmoid—SELU—Tanh
Batch size	1 to 128
Dropout rate	0.1 to 0.5
Number of dense nodes	32 to 1024
Gradient descent optimizer	Adam—Nadam—AdaMax—RMSProp—SGD

**Table 3:** Parameters and values of the five pre-trained CNNs used in this study.

Models	Input Size	Optimizer	Learning Rate	Batch Size
VGG16	224 × 224	Adam	5 × 10^−5^	32
VGG19	224 × 224			
ResNet50	200 × 200			
InceptionV3	150 × 150			
DenseNet201	200 × 200			

**Table 4:** The top five most accurate models.

Trial Number	Activation	Batch Size	Dropout	Dense Nodes	Optimizer	Accuracy	Best Epoch	Time (s)
17	Tanh	1	0.25	32	SGD	98.70	11/30	114
38	ReLU	1	0.24	32	Nadam	98.70	21/30	350
16	Tanh	1	0.25	32	SGD	98.37	16/30	170
12	ReLU	23	0.28	156	Adamax	98.37	18/30	14
15	Tanh	1	0.25	32	SGD	98.05	19/30	204

**Table 5:** Summary of the optimized model.

Layer Type		Kernel Attribute	Number of Filters	Feature Map Size
Image Input Layer				64 × 64 × 1
Main Block 1	Convolutional Layer	3 × 3 × 1, stride 1, padding = same	32	64 × 64 × 32
	Tanh Layer			64 × 64 × 32
	Max-Pooling Layer	2 × 2, stride 2, no padding		64 × 64 × 32
Main Block 2	Convolutional Layer	3 × 3 × 32, stride 1, padding = same	64	32 × 32 × 64
	Tanh Layer			32 × 32 × 64
	Max-Pooling Layer	2 × 2, stride 2, no padding		16 × 16 × 64
Main Block 3	Convolutional Layer	3 × 3 × 64, stride 1, padding = same	64	16 × 16 × 64
	Tanh Layer			16 × 16 × 64
	Max-Pooling Layer	2 × 2, stride 2, no padding		8 × 8 × 64
Main Block 4	Convolutional Layer	3 × 3 × 64, stride 1, padding = same	128	8 × 8 × 128
	Tanh Layer			8 × 8 × 128
	Max-Pooling Layer	2 × 2, stride 2, no padding		4 × 4 × 128
Main Block 5	Convolutional Layer	3 × 3 × 128, stride 1, padding = same	256	4 × 4 × 256
	Tanh Layer			4 × 4 × 256
	Max-Pooling Layer	2 × 2, stride 2, no padding		2 × 2 × 256
Classification Block	Fully Connected Layer			32
	Tanh Layer			
	Dropout			
	Fully Connected Layer			3
	Softmax			

**Table 6:** Comparison between the performance of the scratched model before and after optimization.

Metric	Before Optimization	After Optimization
Validation accuracy	91.88%	98.70%
Training time (s)	117	99

**Table 7:** The effect of reducing the training data on the performance of the optimized model.

Training Data	Validation Data	Accuracy	Loss	Precision	Recall	F1-Score
90%	10%	98.70	0.069	98.33	98.66	98.66
80%	20%	97.24	0.107	97	97	97
70%	30%	97.07	0.095	97	97	97
60%	40%	95.28	0.162	95	95	95
50%	50%	93.17	0.202	93	92	92.66

**Table 8:** Comparative results based on precision, recall, F1-score, accuracy, and loss.

Models	Labels	Precision	Recall	F1-Score	Accuracy	Loss
VGG16	Glioma	0.99	0.96	0.97	0.9708	0.1042
	Meningioma	0.91	0.97	0.94		
	Pituitary	1.00	0.99	0.99		
	Average	0.966	0.973	0.966		
VGG19	Glioma	0.99	0.97	0.98	0.9643	0.1240
	Meningioma	0.92	0.93	0.92		
	Pituitary	0.96	0.99	0.97		
	Average	0.956	0.963	0.956		
ResNet50	Glioma	0.87	0.77	0.81	0.8929	0.2698
	Meningioma	0.59	0.57	0.58		
	Pituitary	0.82	0.98	0.89		
	Average	0.886	0.89	0.89		
InceptionV3	Glioma	0.94	0.97	0.95	0.9286	0.2830
	Meningioma	0.92	0.82	0.87		
	Pituitary	0.92	0.96	0.94		
	Average	0.926	0.916	0.92		
DenseNet201	Glioma	0.97	0.97	0.97	0.9481	0.2081
	Meningioma	0.92	0.85	0.88		
	Pituitary	0.93	1.00	0.96		
	Average	0.94	0.94	0.936		
The optimized CNN	Glioma	0.99	0.99	0.99	0.9870	0.0692
	Meningioma	0.97	0.98	0.98		
	Pituitary	0.99	0.99	0.99		
	Average	0.983	0.986	0.986		

**Table 9:** Accuracy comparison among the optimized CNN model and state-of-the-art methods.

Method	Accuracy
Cheng et al. [10]	91.28
Paul et al. [11]	90.26
N. Abiwinanda et al. [15]	84.18
Swati Z.N. K. et al. [14]	94.82
Hossam H Sultan et al. [17]	96.13
The optimized CNN	98.70

## Data Availability

Not applicable.
